# Experimental Study on the Effectiveness of Polyurethane Flexible Adhesive in Reduction of Structural Vibrations

**DOI:** 10.3390/polym12102364

**Published:** 2020-10-15

**Authors:** Natalia Lasowicz, Arkadiusz Kwiecień, Robert Jankowski

**Affiliations:** 1 Faculty of Civil and Environmental Engineering, Gdansk University of Technology, 80-233 Gdansk, Poland; jankowr@pg.edu.pl; 2 Faculty of Civil Engineering, Cracow University of Technology, 31-155 Cracow, Poland; akwiecie@pk.edu.pl

**Keywords:** polymer adhesive, dynamic load, structural vibrations, damping ratio

## Abstract

The aim of the present study is to consider the idea of using polyurethane flexible adhesive in to reduce the vibrations in structures exposed to dynamic loads and evaluate their damping properties in relation to large deformations. Firstly, two aluminium cantilever beams, simulating structural elements (without and with polyurethane layer in the form of tape), were analysed, in order to check the damping of the unconstrained polymer layer. In the second stage of the study, a composite beam consisting of two aluminium flat beams bonded with polymer adhesive was considered, so as to check the damping of the constrained polymer layer. Dynamic parameters, such as modes of free vibrations, corresponding natural frequencies and damping ratios, were determined and compared. The third stage of the investigation was aimed at solving the problem of the additional mass of the applied polymer layer, which influences the frequencies and damping of the tested structure. A special separating procedure is proposed that makes it possible to calculate the corrected real values of the polymer layer’s damping. The results of the study clearly show that the response of the composite aluminium beam with and without polymer adhesive layer is mainly influenced by the layers’ thickness and the large strain deformation, in terms of its damping characteristics. The use of polymer adhesive layers in constrained and unconstrained conditions leads to a significant reduction in the vibrations of tested beams, while preserving their stiffness at nearly the same level. The applied analysis procedure made it possible for us to separate the damping properties of the analysed polymer layers and evaluate them independently with respect to the influence of integrated structural elements on damping.

## 1. Introduction

Excessive structural vibrations due to different dynamic loads are among the most serious and dangerous situations that can occur in the case of civil engineering structures [1,2,3]. Wind, earthquake or crowd load effects determine the design procedure of structures that are regularly subjected to such significant dynamic loads (see, for example, [4,5,6]). The excited structures are more resistant to dynamic loads when they are characterized by high ductility and damping properties. The use of composite materials in the strengthening of various kinds of structures (infilled reinforced concrete frames or masonry) is nowadays a popular approach [7,8,9,10,11,12]. Composite materials are usually bonded to the structures using stiff adhesives made of mineral mortars or epoxy resins. Such kind of stiff adhesives of brittle behaviour cause stiffening of the strengthened structures and do not introduce ductile and damping properties. Moreover, strengthening of masonry and concrete structures using composite systems on stiff adhesives is not fully effective because of the low strength of the substrates [13]. Stress concentrations overcome strength of the substrates generated by stiff adhesives [14]. A solution to this problem might be the use of polyurethane flexible materials, which are more compatible with masonry and concrete substrate [15,16] and ensure a high amount of ductility and deformation capacity [17].

When the induced vibrations excite a natural frequency of the affected part of the structure, resonance might occur. This may lead to severe damages or even total structural collapse [18,19]. The dynamic characteristics of structures, such as natural frequencies and damping, play important roles in dynamic response of structures, and thus various methods of determination of these characteristics are exploited. Dynamic detection methods are also used in determination of structural condition of structures, including their damage [20,21,22]. Anyway, the application of innovative strengthening or repair solutions in masonry or infill structures, such as composite materials externally bonded [23] or injection filling of cracks [24], also requires increasing the structural damping. This aspect is especially important in seismic areas. Innovative solutions using dissipative viscoelastic elastomers, like PolyUrethane Flexible Joints (PUFJ) and Fibre-Reinforced PolyUrethane (FRPU) [18], manifest efficiency in structural damping, but cause problems in determination of damping properties of polymeric materials separately. Meanwhile, the damping values are required for the dynamic numerical analysis of repaired/strengthened structures, where appropriate damping properties of component materials are sought. Moreover, elastomeric materials usually show nonlinear behaviour, described by hyper elastic theory [25,26], which does not include damping, thus this parameter must be determined experimentally.

Masonry and reinforced concrete structures undergo reduction of stiffness through crack development during earthquakes, resulting in a shift in their natural frequencies [27]. The most commonly used method of vibration attenuation in the resonance range is the application of dampers so as to increase dissipation of energy during vibrations. A few types of such elements (passive, active, half-active and hybrid) are used as an effective method in reduction of structural vibrations [28]. The passive method is often used in such structures as bridges, footbridges, tall buildings, masts, towers and chimneys.

A viscoelastic damper is an example of a passive vibration reduction method [29]. This can be a viscous damper, consisting of a cylinder filled with a highly viscous liquid, in which a piston with holes moves and energy is dissipated as a result of friction between fluid particles and the piston. Another example of a passive damper is the Tuned Mass Damper—TMD (also called vibration absorber or vibration damper)—where an additional mass is attached to a specific location of a structure, so as to reduce the amplitude of vibrations to an acceptable level during dynamic excitation. This type of passive damper is used to reduce vibrations of structures such as footbridges, bridges and tall buildings [30,31]. The most famous examples of using TMD systems include the Millennium footbridge in London and the Taipei skyscraper in Taiwan.

The alternative idea of using polymer material, which has already been successfully applied for filling cracks in order to repair damaged masonry structures [27,32], was considered in this study. The analysed polymer adhesive is a specially designed flexible two-component grout, which is based on polyurethane resin [33,34]. It has been confirmed, based on previous experimental studies (see [35,36]), that the material also has additional damping properties. Moreover, it is characterized by very good adhesion to steel and concrete and high deformability [37]. Good damping properties of polyurethane have also been confirmed during shaking table tests, in which a damaged infill specimen was protected by externally bonded composite GFRP strengthening using flexible polyurethane adhesive (FRPU) [18]. The application of this solution allowed the weakened structure to survive dynamic excitation in the range of resonance. Only damping properties of the deformable elastomeric adhesive of the FRPU protected the structure against collapse, when the tested structure manifested large displacements (up to 10 cm), generating large deformations in the elastomeric adhesive layers.

The numerical analysis of the tested dynamic system requires knowledge about damping properties of the flexible adhesive, and thus experiments and analysis should be focused on determining the damping of a single polyurethane layer. This problem is not easy to solve, because the influence of large strains in the deformed adhesive layer has to be taken into account. Moreover, boundary conditions applied to the elastomeric layer, as well as the thickness of the layer, are of great importance. Moreover, to make analysis as simple as possible, polymeric adhesive layers can be combined in testing with materials of well-known and stable parameters, like aluminium [26,38] or glass [39,40]. Such composite structures can be easily tested, and parameters of elastomeric layers can be determined indirectly [41].

In this paper, the results of experimental investigation, concerning the effectiveness of polymer adhesive in reduction of vibrations, are described. The experiment has been divided into two parts. In the first stage, two aluminium cantilever beams (without and with polymer layer in the form of a tape) have been analysed. The second stage of the study focuses on five cantilever composite beams consisting of two aluminium flat bars bonded with polymer adhesive of different thickness (see also [26,38]). The specimens were forced to induce large deflections of the beams and thus large strain deformations of the polymer layers. In the analysis, changes in thickness and in shear strain were considered. Representative impact load cases, induced by using a modal hammer, are presented and described in detail in the paper. Dynamic parameters, such as modes of free vibrations, corresponding values of natural frequencies and damping ratios, were determined. Next, a detailed analysis, focused on the interpretation of the results, was carried out using the approaches described in [18,26,37]. Moreover, a new approach, separating the influence of equivalent stiffness and additional mass on damping of a polymeric layer, is also proposed. Large shear deformation and thickness of an adhesive have been investigated as factors influencing damping properties.

## 2. Investigation on Plain and Composite Aluminium Beams with Polymer Layer

### 2.1. Experimental Study—Part 1

#### 2.1.1. Experimental Setup

**Figure 1:**
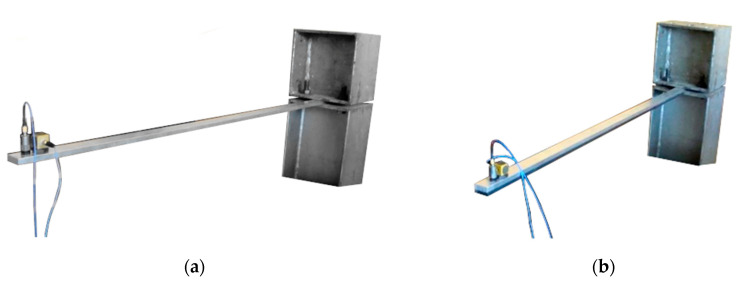
(**a**) Aluminium cantilever beam; (**b**) aluminium cantilever beam with polymer adhesive.

The first stage of the investigation was devoted to the analysis of two aluminium cantilever beams with a total length of 965 mm, a width of 30 mm and a height of 9 mm. One of them represents a plain cantilever beam (see Figure 1a), while the second one considers a beam with a polymer layer applied in the form of tape of the same width as the beam, with a total thickness of 7 mm, located on the bottom of the beam (see Figure 1b).

**Figure 2:**
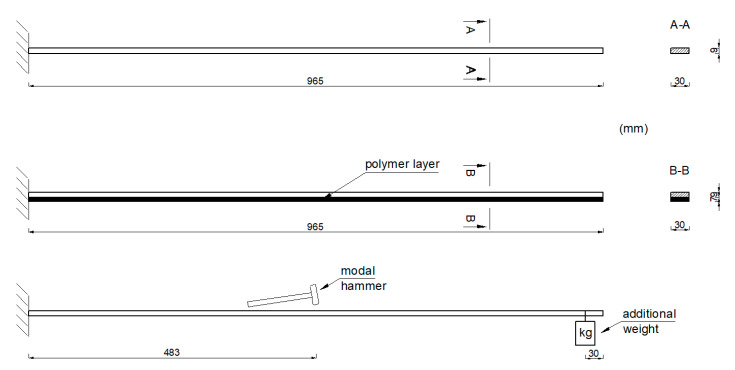
Schematic diagrams of aluminium cantilever beams (without and with the polymer layer).

The study focused on determining the effectiveness of the polymer layer for reducing the structural vibrations at various levels of large strain deformation. Dynamic parameters, such as modes of free vibrations and the corresponding natural frequencies of the two aluminium cantilever beams (without and with the polymer layer) were estimated. The aluminium cantilever beams were induced to vibrate by impacts with a modal hammer applied in the middle of the elements’ length. Schematic diagrams of both of the analysed beams and the manner in which they were induced to vibrate are presented in Figure 2. A modal hammer type PCB 086C01 (PCB Piezotronics, Inc., Depew, NY, USA), with a force that was variable over time and a maximum value of *F* = 15 N, was used. The total duration of each measurement was set to be equal to 12.5 s. The behaviour of the beams was observed and recorded using two accelerometers, including a triaxial one (PCB Piezotronics, Inc., Depew, NY, USA), that were installed on the metal side at the end of each beam. The response of the cantilever beams was analysed under various values of vertical load (weights with a mass ranging from 1 kg to 6 kg), applied in a distance of 30 mm from their ends (see Figure 2). This applied load resulted in large deflections of the beams and their visible curvature.

The aluminium used in this study possessed the following parameters: Young’s modulus *E* = 70 GPa, Poisson’s ratio ν = 0.3, and mass density ρ = 2700 kg/m^3^ (producer data). In the case of the polyurethane layer, which was made of Sika PSM (SIKA Poland, Cracow, Poland), the properties of the material were: Young’s modulus *E* = 6 MPa, Poisson’s ratio ν = 0.48, mass density ρ = 1000 kg/m^3^, tensile strength *R*_m_ = 2.5 MPa, shear strength *R*_t_ = 1.2 MPa, and ultimate strain ε = 110% (producer data).

#### 2.1.2. Experimental Results

**Figure 3:**
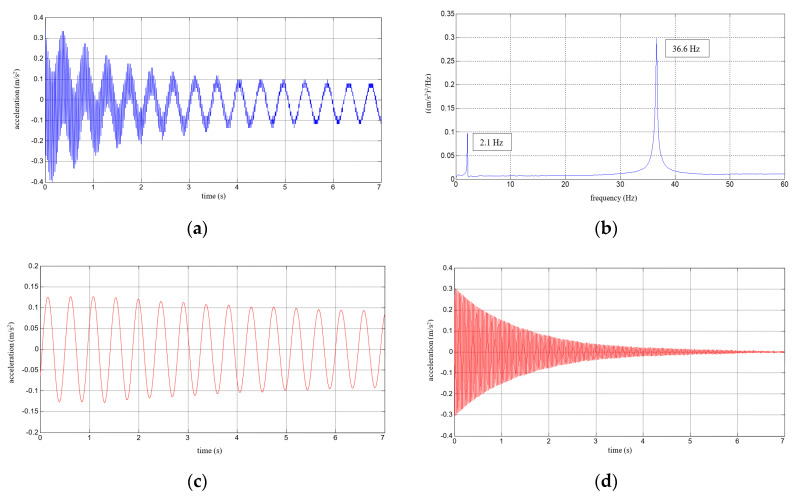
Results for an aluminium cantilever beam: (**a**) acceleration time history; (**b**) Fourier Spectrum; (**c**) vibrations with the 1st natural frequency; (**d**) vibrations with the 2nd natural frequency.

**Figure 4:**
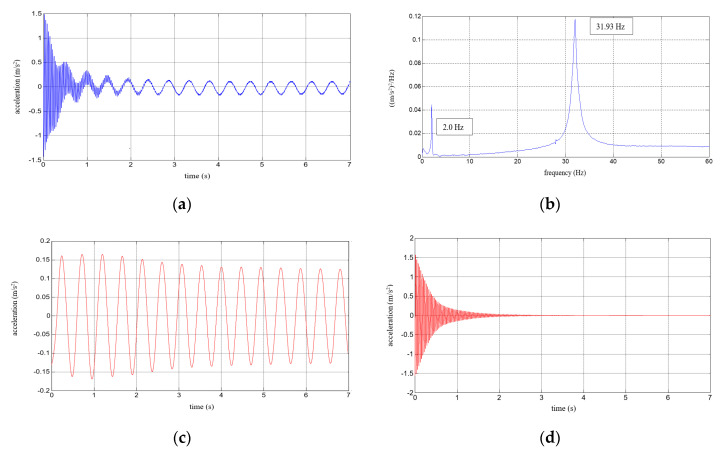
Results for an aluminium cantilever beam with polymer layer: (**a**) acceleration time history; (**b**) Fourier Spectrum; (**c**) vibrations with the 1st natural frequency; (**d**) vibrations with the 2nd natural frequency.

**Figure 5:**
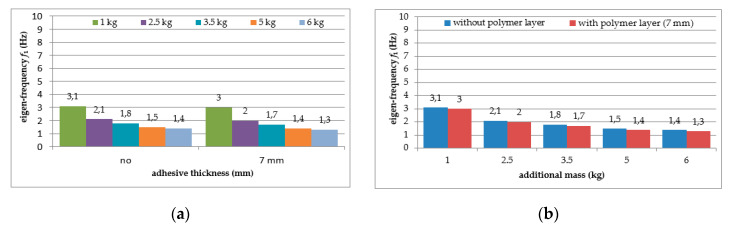
Comparison of first natural frequency values in relation to: (**a**) loading mass change; (**b**) presence of polymer layer.

**Figure 6:**
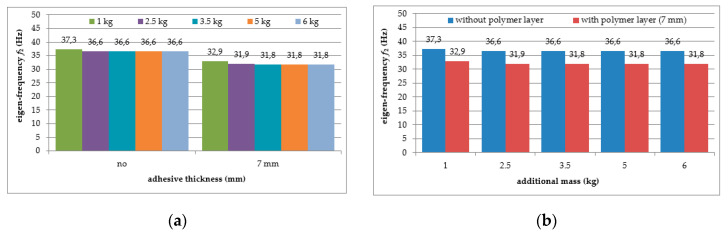
Comparison of second natural frequency values in relation to: (**a**) presence of polymer layer; (**b**) loading mass change.

Six repetitions of each measurement under each load case were conducted, and the mean values of the natural frequencies corresponding to the first two modes of free vibration, as well as the mean values of the damping ratios, were estimated. The results of the experimental study were determined in the form of acceleration time histories, based on which modal characteristics were obtained. Two representative acceleration time histories, describing the behaviour of the aluminium cantilever beams (without and with polymer layer) with an additional weight of 2.5 kg are presented in Figure 3a and Figure 4a, respectively. Two modes of free vibrations and the corresponding natural frequencies for each were determined using Fast Fourier analysis (see Figure 3b and Figure 4b). Both natural frequencies observed during experimental study were analysed separately by filtering out the components with other frequencies from the measured acceleration time histories. The acceleration time histories of vibrations with the 1st natural frequency (2.1 Hz and 2.0 Hz, respectively) are presented in Figure 3c and Figure 4c. Moreover, Figure 3d and Figure 4d show the results of vibrations with the 2nd natural frequency (36.6 Hz and 31.93 Hz, respectively). The mean values of the natural frequencies calculated for both aluminium cantilever beams are also summarized in Table 1. The values presented there in brackets indicate the frequency change ratio with respect to the first and the second natural frequency values of the specimen loaded with an additional mass of 1 kg, without the polymer layer. Moreover, comparisons of the first and the second natural frequency values are presented graphically in Figure 5 and Figure 6, respectively, with respect to the presence of the polymer layer and changes in loading mass.

As can be seen from Table 1, a significant decrease in the natural frequency of the aluminium cantilever beam can be observed for the first natural frequency as a result of the application of an additional vertical load in the form of weights with different mass (causing large deformations of the tested structure). The reduction reaches value up to 55% in a slightly nonlinear manner, and is strongly related to the change in the loading mass from 1 kg to 6 kg—see Figure 5a. This observation is valid for both of the analysed beams (with and without the polymer layer) and no significant differences in the frequencies were observed for both beams—see Figure 5b. On the other hand, the second natural frequency was practically insensitive to the mass change (with a reduction of up to only 2% for the plain beam and up to only 4% for the polymer bonded beam)—see Figure 6a. The results also indicate that the application of an additional polymer layer with a thickness of 7 mm led to a reduction in the second natural frequency of up to 13% (independent of the increase in mass)—see Figure 6b.

In the next step of the analysis, the damping ratio was estimated for each case so as to quantify the effectiveness of polymer for reducing structural vibrations. The mean values of damping ratios for the aluminium cantilever beams (without and with the polymer layer) were separately determined for both frequencies of natural vibrations for the loaded beams. The damping ratio, *ζ*, was calculated according to the following formulas (see [1]):

$$\delta =\frac{1}{n}\cdot \sum_{i=1}^{n}\ln\left(\frac{a_{j}}{a_{j+1}}\right),$$

$$\zeta =\frac{\delta }{2\pi n},$$

$$\zeta =\frac{1}{2\pi n}\cdot \left[\frac{1}{n}\cdot \sum_{i=1}^{n}\ln\left(\frac{a_{j}}{a_{j+1}}\right)\right],$$

where δ = logarithmic decrement; *n* = number of cycles; *a_j_* = amplitude of *n*-th cycle; *a_j_*_+1_ = amplitude of (*n* + 1)-th cycle.

**Figure 7:**
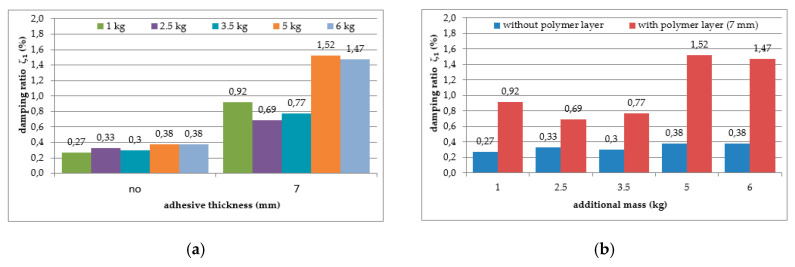
Comparison of damping ratio values for the first natural frequency in relation to: (**a**) presence of polymer layer; (**b**) loading mass change.

**Figure 8:**
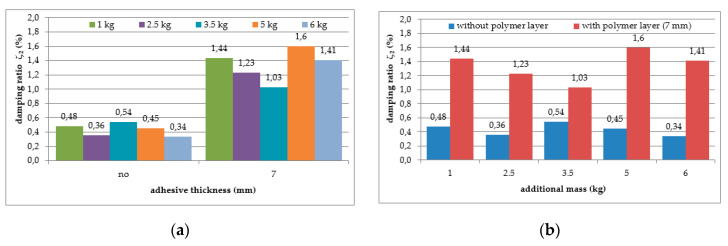
Comparison of damping ratio values for the second natural frequency in relation to: (**a**) presence of polymer layer; (**b**) loading mass change.

The mean values of damping ratios, estimated for the plain aluminium cantilever beam and for the beam with the polymer layer, are summarized in Table 2. The values presented there in brackets indicate the change in damping ratio with respect to the damping ratios determined for the first and the second natural frequency of the specimen loaded with 1 kg additional mass and without the polymer layer. Additionally, graphical comparisons of the damping ratio values for the first and the second natural frequency are shown in Figure 7 and Figure 8, respectively, with respect to presence of the polymer layer and the change in loading mass.

As can be seen from Table 2, an irregular increasing trend could be observed in the damping ratio of the aluminium cantilever beams with and without polymer layer for both analysed natural frequencies due to the application of additional vertical load in the form of weights with different mass—see Figure 7a and Figure 8a. Irregular increases and decreases were observed in all analysed cases. The application of an additional polymer layer with a thickness of 7 mm caused a significant increase in the damping ratio for both analysed natural frequencies—see Figure 7b and Figure 8b. The damping ratio was even increased by up to 2–4-fold for the first and the second natural frequencies, but no regularity was observed with respect to increasing mass of additional weights.

#### 2.1.3. Discussion of Results Obtained

The results of the first experiment confirm that the application of the polymer layer causes practically no shift in the first natural frequency of the cantilever beam (see Table 1 and Figure 5b), but for the second one, a reduction of 13% was observed (Figure 6b). This fact indicates that the damping properties of a structure can only be improved in the higher frequency ranges by externally bonding a polymer layer to only one side of the structure. The nonlinearly proportional reduction in the first natural frequency with the load (mass) increase (Figure 5a) is obvious, but the lack of reaction with the change in load in the case of the second frequency (Figure 6a) could be caused by the non-proportional mass distribution (i.e., it was located at the cantilever end). However, changes in the first frequency were strongly dependent on additional mass; thus, deeper analysis of this aspect will be provided later in the interpretation section.

Moreover, the application of the polymer layer led to a visible increase in the values of the structural damping ratio of the aluminium cantilever beams (see Table 2 and Figure 7 and Figure 8). Damping, as well as frequency, is dependent on additional mass. This aspect must be considered in the analysis. Additionally, no clear correlation was observed between the damping ratio and the load increase, even though the values of damping ratio were, generally speaking, higher for larger values of mass in the case of the 1st natural frequency. The influence of this additional mass on damping properties is discussed later in the interpretation section.

In any case, the above comparison clearly shows that the applied polyurethane Sika PSM exhibited a significant influence on the damping properties of the beam with respect to the element when no polymer layer had been applied. The presence of the polyurethane adhesive increased the damping ratio 2–5-fold in the cases of low (1–3 Hz) and high (30–38 Hz) frequencies, when comparing cases with the same load. An open issue is the influence of the thickness of the polymer layer and the increase of the additional mass (causing an increase in the large deflection of the cantilever beam, thus generating an increase in shear strain in the polymer in the range of large deformations).

### 2.2. Experimental Study—Part 2

#### 2.2.1. Experimental Setup

**Figure 9:**
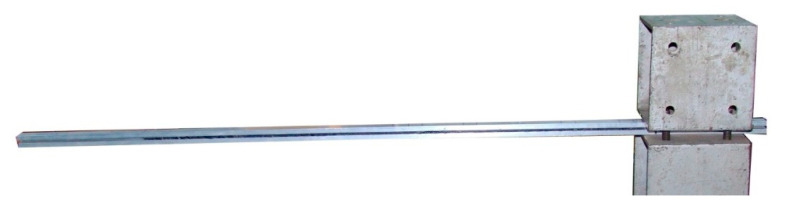
Aluminium cantilevered composite beam.

In the next stage of the experimental study, composite cantilever beams consisting of two aluminium flat bars with a total length of 1250 mm, a thickness of 9 mm, and a width of 30 mm, bonded together for a length of 992 mm with polymer adhesive of different thicknesses (0.5 mm, 1.2 mm, 1.75 mm, 3.1 mm and 5 mm) were analysed—see Figure 9. The investigation was conducted so as to verify the effectiveness of the application of polymer adhesive for the reduction of vibrations in the aluminium cantilever beam when the polymer layer is constrained on both sides by structural elements. For this purpose, dynamic parameters such as the modes of free vibrations and the corresponding natural frequencies were determined. Similar to the case of the previous tests, the aluminium cantilever beams were induced to vibrate through impacts with a modal hammer applied at the middle of the elements’ length. The total time of each measurement was also equal to 12.5 s. The tests included measurements carried out for beams with additional vertical loads applied at their ends in the form of weights with a mass of 1 kg, 2.5 kg, 3.5 kg, 5 kg and 6 kg. The material properties of the aluminium and polymer adhesive used during the experimental study are described in Section 2.1.

#### 2.2.2. Experimental Results

**Figure 10:**
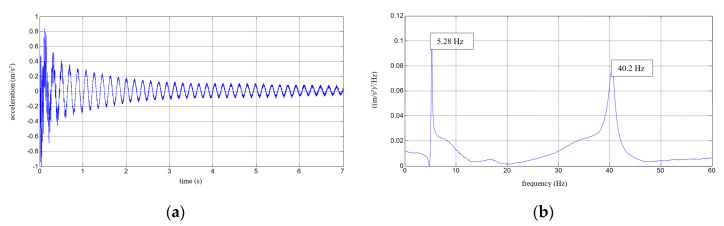

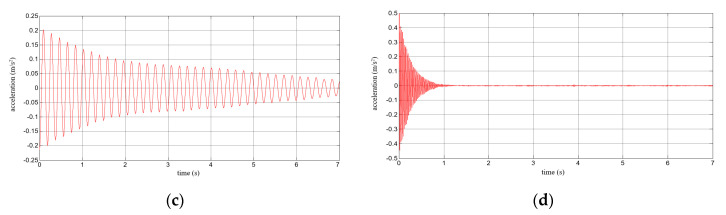
Results for a composite aluminium cantilever beam with 0.5 mm thick polymer adhesive: (**a**) acceleration time history; (**b**) Fourier Spectrum; (**c**) vibrations with the 1st natural frequency; (**d**) vibrations with the 2nd natural frequency.

**Figure 11:**
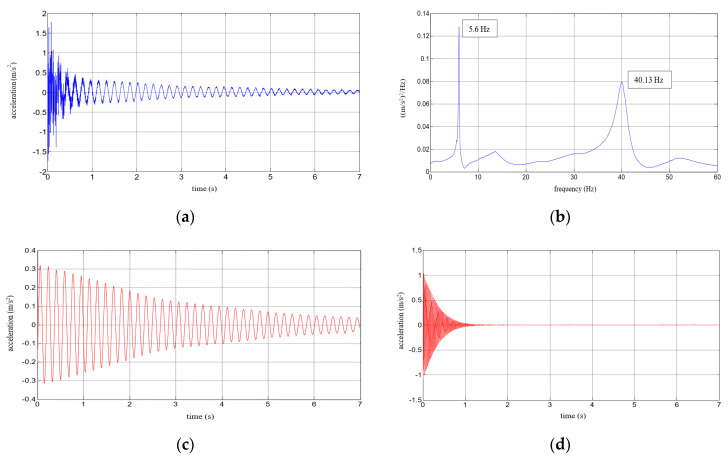
Results for a composite aluminium cantilever beam with 1.2 mm thick polymer adhesive: (**a**) acceleration time history; (**b**) Fourier Spectrum; (**c**) vibrations with the 1st natural frequency; (**d**) vibrations with the 2nd natural frequency.

**Figure 12:**
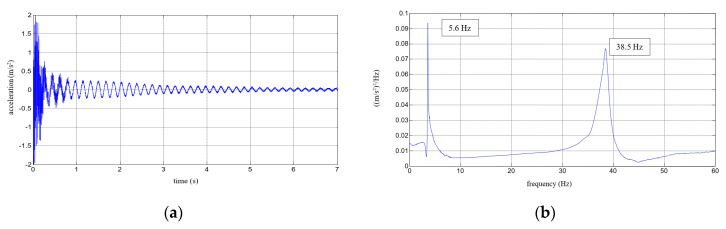

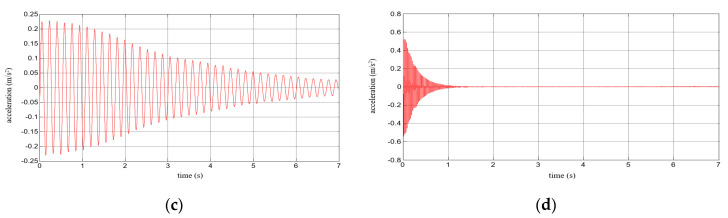
Results for a composite aluminium cantilever beam with 1.75 mm thick polymer adhesive: (**a**) acceleration time history; (**b**) Fourier Spectrum; (**c**) vibrations with the 1st natural frequency; (**d**) vibrations with the 2nd natural frequency.

**Figure 13:**
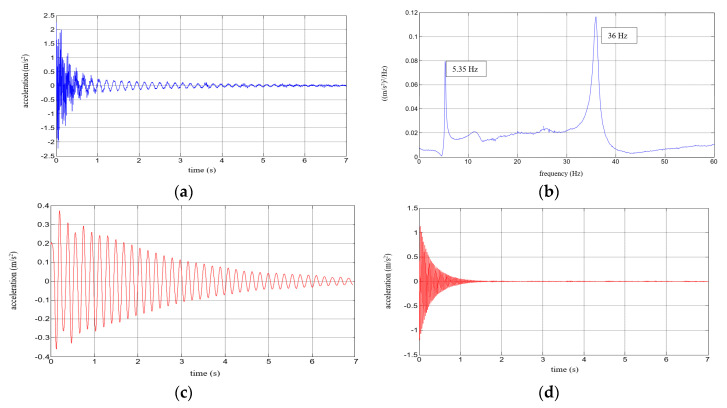
Results for a composite aluminium cantilever beam with 3.1 mm thick polymer adhesive: (**a**) acceleration time history; (**b**) Fourier Spectrum; (**c**) vibrations with the 1st natural frequency; (**d**) vibrations with the 2nd natural frequency.

**Figure 14:**
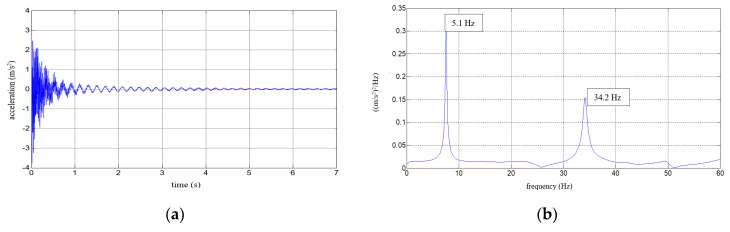

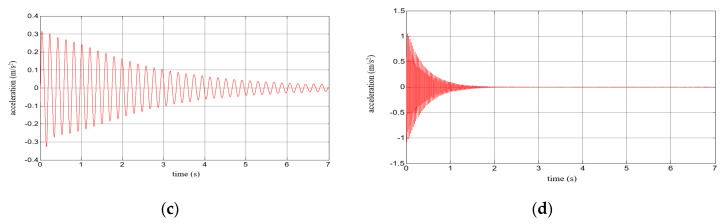
Results for a composite aluminium cantilever beam with 5 mm thick polymer adhesive: (**a**) acceleration time history; (**b**) Fourier Spectrum; (**c**) vibrations with the 1st natural frequency; (**d**) vibrations with the 2nd natural frequency.

Six repetitions were carried out for each measurement under each load. The results of the experimental study were determined in the form of acceleration time histories, based on which the modal characteristics, including mean values of natural frequencies corresponding to the first two modes of free vibrations and the mean values of damping ratios, were determined. Five representative acceleration time histories describing the behaviour of a composite aluminium cantilever beam (with polymer adhesive with thicknesses of 0.5 mm, 1.2 mm, 1.75 mm, 3.1 mm and 5 mm) with an additional weight of 1 kg are presented in Figure 10a, Figure 11a, Figure 12a, Figure 13a and Figure 14a. Two modes of free vibrations and corresponding natural frequencies for each case were determined by conducing Fast Fourier analysis (see Figure 10b, Figure 11b, Figure 12b, Figure 13b and Figure 14b). Both natural frequencies observed during experimental study were analysed separately by filtering out the components with other frequencies from the measured acceleration time histories. The acceleration time histories of the vibrations with the 1st natural frequency are presented in Figure 10c, Figure 11c, Figure 12c, Figure 13c and Figure 14c. In Figure 10d, Figure 11d, Figure 12d, Figure 13d and Figure 14d, the results of the vibrations with the 2nd natural frequency are shown.

**Figure 15:**
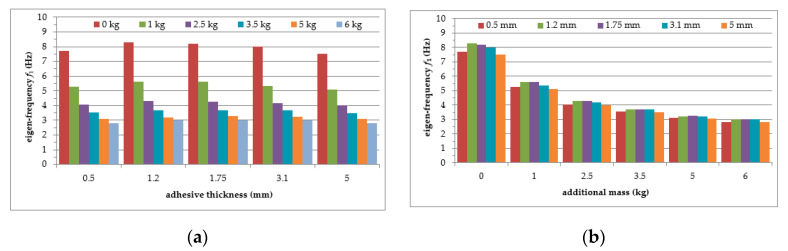
Comparison of first natural frequency values in relation to: (**a**) polymer adhesive thickness; (**b**) loading mass change.

**Figure 16:**
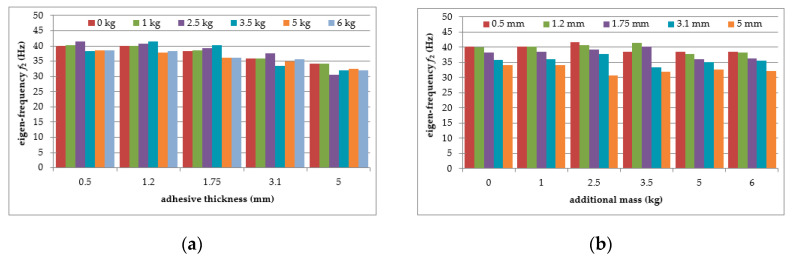
Comparison of second natural frequency values in relation to: (**a**) polymer adhesive thickness; (**b**) loading mass change.

The mean values of the natural frequencies determined for the composite aluminium cantilever beams are summarized in Table 3. The comparison of the first and second natural frequency values with respect to the presence of the polymer layer and change in loading mass change estimated for the beams is presented graphically in Figure 15 and Figure 16, respectively.

As can be seen from Table 3, a significant decrease in the natural frequency of the aluminium cantilever beam can be observed for the first natural frequency, due to the application of an additional vertical load, as was observed in part 1 of the experimental study. The reduction of nonlinear behaviour reaches 64%, and this is strongly related to the change in the loading mass from 1 kg to 6 kg—see Figure 15a. This observation is valid for all of the analysed beams (with polymer layers of different thickness). Small differences in frequencies were observed for the beams with changing layer thickness—see Figure 15b. The second natural frequency was slightly dependent on the mass change (with up to a 6% reduction for the beams with the maximum additional mass)—see Figure 16a. The change in polymer layer thickness did not exhibit a clear relationship with the changes in the second natural frequency—see Figure 16b.

**Figure 17:**
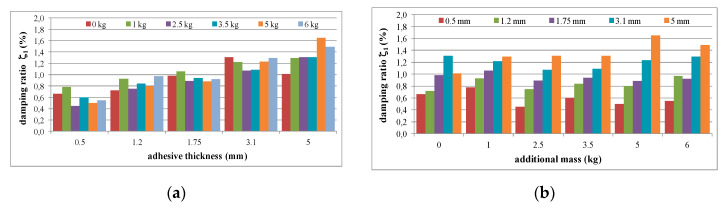
Comparison of damping ratio values for the first natural frequency in relation to: (**a**) polymer adhesive thickness; (**b**) loading mass change.

**Figure 18:**
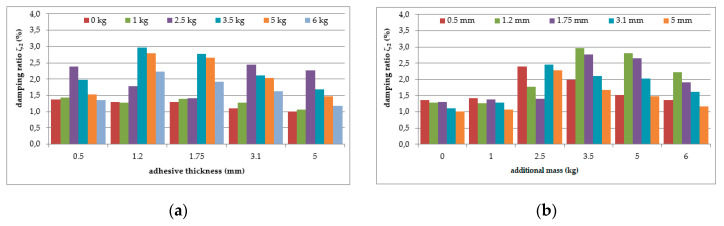
Comparison of damping ratio values for the second natural frequency in relation to: (**a**) polymer adhesive thickness; (**b**) loading mass change.

The mean values of the damping ratios with respect to the natural frequencies determined (as previously) for the composite aluminium cantilever beams are summarized in Table 4. The comparison of the damping ratio values for the first and second natural frequency with respect to the presence of the polymer layer and the change in loading mass estimated for the beams is presented graphically in Figure 17 and Figure 18, respectively.

As can be seen from Table 4, a small, almost regular increasing trend (up to 1.5%) could be observed in the damping ratio of the analysed aluminium cantilever beams for the first natural frequency as a result of the changes in polymer layer thickness—see Figure 17a. Irregular increases and decreases were observed as a result of additional mass changes—see Figure 17b. The damping ratio determined for the second natural frequency was strongly irregular, and no clear relation could be observed with the increase in mass of the additional weights and the changes in the thickness of the polymer layer—see Figure 18.

#### 2.2.3. Discussion of the Results Obtained

The results of the second experiment show that the application of additional vertical load in the form of weights of varying mass led to a significant decrease in the 1st natural frequency (*) of the composite beam with polymer adhesive layers of various thickness (see Table 3 and Figure 15a). On the other hand, only a small lowering of the frequencies was observed for the 2nd natural frequency (**) (changes varied +/− 10%—see Table 3 and Figure 16a). Moreover, the application of thicker polymer layers decreased the natural frequency of the composite beam by 6% in the case of the 1st mode of free vibrations (*), and by 15–26% in the case of the 2nd mode of free vibrations (**), as can be observed from Table 3 and Figure 15b and Figure 16b. However, it must be noted that the results for thinner polymer layers deviated from this pattern.

The application of a thicker polymer layer led, generally speaking, to an increase in the structural damping ratios of the composite beams in the case of the 1st natural vibration mode (see Table 4 and Figure 17a). The highest increase (by 230%) was observed for the beam with an additional mass of 5 kg. On the other hand, the increasing trend of the structural damping ratios was not so clear in the case of the 2nd natural vibration mode (see Table 4 and Figure 18a). For this mode, the highest increase (by 84%) in the damping ratio was recorded for the beam with a load of 5 kg. It can also be seen from Table 4 and Figure 17b and Figure 18b that the change in the damping ratios determined for composite beams with additional loads in the form of weights with different mass did not really show any decreasing or increasing trend. In the case of the 1st natural frequency (*), for example, the damping ratio decreased by up to 34% and increased by up to 63%, without any apparent rule.

It should be mentioned that the investigation presented here is a simulation of the functioning of flexible polyurethane adhesive between a structural substrate (concrete, masonry) and a composite laminate. Large shear deformation of the adhesive layer is permitted due to the deflection of the cantilever beam, where the polymer interface undergoes shear between the two aluminium elements. Larger loads at the cantilever end generate a greater bending moment, which increases the nonlinearity of the flexible polyurethane adhesive behaviour by increasing of shear strain of the polymer layer. Using data presented in [24], the shear strain calculated for the analysed polymer layers reached a value of 0.3, which places the obtained strain of 30% within the range of large deformations. By comparing the influence of this factor, it can be seen that the increase in deformation (caused by an increase in load) results in an increase in damping only for the highest tested adhesive thickness of 5 mm and for low frequencies. For high frequencies, the highest damping was observed for medium values of deformation. Moreover, higher frequencies were damped to an extent about two times greater than the lower ones.

It seems that the influence of additional mass on the results with respect to frequency and damping is significant, and this makes the simple interpretation of damping relations introduced by the polymer layer difficult due to its nonlinear behaviour. For this reason, an additional analysis, presented in the next section, was carried out, aiming to separate out the damping relationships for the polymer layer, such that they are independent from the interference due to the application of additional mass. The corrected results for damping were interpreted in relation to the change in polymer layer thickness and the increase in large deformations.

## 3. Approach for Correction of Results

It is well known that a change in mass in a dynamically analysed system will influence the natural frequency values. Assuming that the tested cantilever beams are systems with evenly distributed structural mass, and taking into consideration only the first natural frequency (additional mass at the cantilever end mainly influences the first natural mode), a procedure for the determination of damping with reference to additional mass is proposed. The first natural frequency of the assumed system can be calculated using the well-known Equation (1).

(1) $$f_{1}=\frac{3.516}{2\pi L^{2}}\sqrt{\frac{EI}{\mu }}$$

where: *L*—length of the cantilever beam [m]; *EI*—bending stiffness of the cantilever beam [Nm^2^]; μ—mass of the cantilever beam evenly distributed along its length [kg/m].

The change in the first natural frequency is dependent on stiffness *EI* and mass *μ*, whereas the equation part in front of the root can be assumed to be a constant value for the specified length *L*. In the cases of the tests on aluminium cantilever beams with polymer layers described above, the stiffness *EI* of the aluminium beam and a polymer layer can be easily determined: *EI*_A_ = 127.6 Nm^2^ for the aluminium beam (9 mm of thickness), and *EI*_P_ = 0.005 Nm^2^ for the polymer (Sika PSM) layer with a thickness of 7 mm (see experimental study—part 1). If we analyse the cross-section of the beam with the polymer layer (*EI*_AP_ = *EI*_A_ + *EI*_P_) and without it (*EI*_A_), the stiffness ratio *EI*_AP_/*EI*_A_ ≈ 1. The application of thinner polymer layers between the two aluminium bars (see experimental study—part 2) does not change this ratio, which means that the frequencies and damping ratios of the analysed cases are not dependent on the stiffness ratio.

On the other hand, the mass of the analysed systems changes in the cases of the tests on the aluminium cantilever beams with the polymer layers described above. Mass evenly distributed along the aluminium beam length of the plain aluminium beam (μ_A_) and of the aluminium beam with the polymer layer (thickness of 9 mm) (μ_AP_) varies, starting from 1 (see experimental study—part 1). The calculated ratio μ_AP_/μ_A_ influences the frequencies and damping values, and thus the values of damping dependent on it should be selected and introduced as a correction for the determined damping ratios.

The proposed approach of determining the mass influences is based on the ratio of frequencies given by Equation (1), derived for the case described in part 1 of the experimental study. A modification of this approach was used in [18] in order to determine building stiffness degradation on a shake table. A comparison of the mass influences for the plain aluminium beam and the same beam with the polymer layer is derived from Equation (2), assuming *EI*_AP_/*EI*_A_ ≈ 1. This equation links frequency ratio with a mass change factor α, defined as the ratio of evenly distributed masses μ_AP_/μ_A_ = α, when μ_AP_ > μ_A_.

(2) $$\frac{f_{1\mathrm{AP}}}{f_{1A}}=\frac{\sqrt{\frac{EI_{\mathrm{AP}}}{\mu_{\mathrm{AP}}}}}{\sqrt{\frac{EI_{A}}{\mu_{A}}}}\;\;\to \;\;{\left(\frac{f_{1\mathrm{AP}}}{f_{1A}}\right)}^{2}=\frac{\mu_{A}}{\mu_{\mathrm{AP}}}=\frac{1}{\propto }$$

Considering the dynamic characteristic and damping of the system with the first natural frequency described by Equation (1) and assuming the simplification of this system to a single-degree-of-freedom system (by considering the additional mass at the cantilevers’ ends) with the damped free vibrations [42], Equation (3) can be used for the locus of the amplitude maxima in variation with frequency and damping. A simple transformation of Equation (3) with Equation (2) leads to Equation (4), which defines the relation between the change in the damping ratio Δζ and the change in mass from μ_A_ to μ_AP_, described by the mass change factor α.

(3) $$\frac{f_{1}}{f_{1n}}=\sqrt{1-\zeta^{2}}\;\;\to \;\;{\left(\frac{f_{1\mathrm{AP}}}{f_{1A}}\right)}^{2}=1-\zeta^{2}=\frac{1}{\propto }$$

where: *f*_1_—damped frequency [Hz]—lower frequency related to *f*_1AP_; *f*_1n_—natural frequency [Hz]—higher frequency related to *f*_1A_; ζ—damping ratio [-]

(4) $$\Delta \zeta =\sqrt{1-\frac{1}{\propto }}\;\;$$

**Figure 19:**
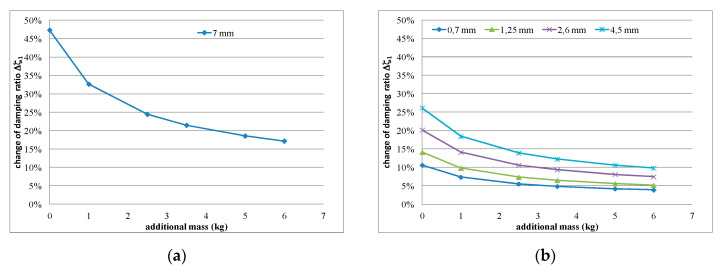
Comparison of the change in damping ratio Δ*ζ* values with the additional load (shear strain of polymer layer) and the increase in polymer layer thickness, presented for the experimental studies: (**a**) part 1 and (**b**) part 2.

Values of *α*, calculated using Equation (2), and Δ*ζ*, calculated using Equation (4), are presented in Table 5 and Table 6 for the specimens tested in part 2 of the experimental study. The changing character of the damping ratio Δ*ζ* is also presented graphically in Figure 19. The presented graphs indicate that the change in damping ratio Δζ is nonlinear in nature, and that it decreases with additional loading mass increase (shear strain increase). Moreover, the thicker the polymer layer, the higher the percentage of change in the damping ratio Δζ. The gradient of change is higher for the unconstrained polymer layer (Figure 19a) than for the constrained one (Figure 19b).

**Figure 20:**
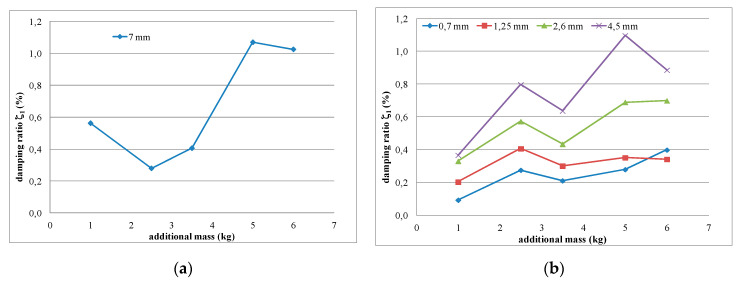
Corrected distributions of damping ratio ζ_1P_ after correction of measured damping values on mass influences, presented for: (**a**) the unconstrained polymer layer (7 mm thickness) and (**b**) various increments of the constrained polymer layer thicknesses.

Figure 20 presents the results of using the proposed procedure. The calculated damping ratio related to the additional increase in polymer mass (ζ_1A_(1+Δζ)) was eliminated from the measured damping ratio values *ζ*_1AP_ according to Equation (5), allowing the damping ratio of the polymer layer ζ_1P_ to be determined. The corrected distribution of the damping ratio ζ_1P_ of the unconstrained polymer layer (7 mm thickness), following correction of the measured damping values (Figure 7b) and in relation to the increase in shear strain, is presented in Figure 20a. Similarly obtained values of ζ_1P_ are shown in Figure 20b for various increments of the constrained polymer layer thicknesses. This figure confirms that the real damping ratio of the polymer layer (corrected by removing the additional mass influence) increases with the increase in the polymer layer thickness, and also increases with the increase in shear strain (additional load causing large deflection).

(5) $$\zeta_{1P}=\zeta_{1\mathrm{AP}}-\zeta_{1A}\left(1+\Delta \zeta \right)$$

## 4. Conclusions

The present paper was devoted to an experimental study focused on the effectiveness of polymer adhesive for the reduction of vibrations in structures exposed to dynamic loads. The experiment was carried out in aluminium cantilever beams, for which the natural frequencies and damping ratios were determined.

In the first stage of the study, two aluminium cantilever beams (without and with polymer layer in the form of tape) were investigated. A significant decrease in the natural frequency of the aluminium cantilever beams, due to the application of an additional vertical load in the form of weights of different mass, was observed. In the case of the frequency corresponding to the 1st mode of free vibrations, an additional mass of 6 kg caused a shift in the natural frequencies to lower values, and the reduction in frequency was found to be as great as 55%, with respect to the case where a mass of 1 kg was applied. Moreover, the results of the study show that the installation of a polymer layer in the form of tape reduces the natural frequency by 3–7% in the case of the 1st natural frequency and by 2–13% in the case of the 2nd natural frequency. The application of the polymer layer leads to an increase in the value of the structural damping ratio by 11–41% in the case of the 1^st^ natural frequency, and does not vary so much (from −29% to +12%) in the case of the 2nd natural frequency. The investigation shows that the applied polyurethane Sika PSM increases the damping ratio significantly (as much as 2–5 times) in the cases of low (1–3 Hz) and high (30–38 Hz) frequencies.

The second stage of the study was focused on a cantilever composite beam consisting of two aluminium flat bars bonded with polymer adhesive of varying thickness. The dynamic parameters, such as mode of free vibrations and corresponding natural frequencies, as well as damping ratios, were estimated. The results of the study indicate that the application of additional load led to a decrease in the values of the 1st natural frequencies for composite beams of up to 61%, while only a small shift in frequencies (changes vary +/− 10%) was observed for the 2nd natural frequencies. Moreover, the application of the polymer layer increased the natural frequency of the composite beams by 6–8% for the 1st natural frequency, and by 15–26% for the 2nd natural frequency. Additionally, the results clearly show that the application of polymer adhesive led to a substantial increase in the damping ratios of the beams (an increase of 230% for the 1st natural frequency and of 84% in the case of the 2nd natural frequency). The investigation shows that the increase in deformations results in a damping increase only for the highest tested adhesive thickness, when considering low frequencies. For high frequencies, the largest damping was observed for middle values of deformation. A significant increase in damping was also observed with the increase of thickness for low frequencies. For higher frequencies, the largest damping was recorded for medium values of adhesive thickness. Higher frequencies were damped about 2 times more than lower ones.

The third stage of the study aimed to solve the problem of the additional mass of the applied polymer layer, which influences the frequencies and damping of the tested structure. It was shown that very large differences in stiffness between the aluminium beams and the bonded polymer layers (4–5 orders) did not influence the global stiffness of the specimen. On the other hand, mass changes influenced the dynamic parameters, causing difficulties in determining the real damping properties of the polymer layer. A special separating procedure was proposed that makes it possible to calculate the corrected real values of the polymer layer’s damping. The distribution of these values, with respect to the layer thickness and large shear strains, confirmed that the increase in the polymer layer thickness results in an increase in damping. The same conclusion was observed in relation to the increase in shear strain, which also caused an increase in damping.

The results obtained from the study indicate that the application of polymer adhesive can be considered an effective method for reducing vibrations in the tested frequency range (up to 40 Hz). Moreover, the simulation of a working composite tape bonded to a substrate using flexible polyurethane adhesive allowed us to recognize that flexible polyurethane adhesives could be effective in damping structural vibrations in the resonance range, where large displacements and large shear strains occur.

The results from the analysis carried out in this paper will be used in numerical analysis, where precise damping properties of applied components are required. We plan to avoid some inaccuracies observed in the results by using modal analysis and the half-band power method, instead of the logarithmic decrement method, for determination of the damping properties.

## Figures and Tables

**Table 1:** Mean values of natural frequencies, corresponding to the 1st (*) and the 2nd (**) mode of free vibrations, calculated for both aluminium cantilever beams (without and with polymer layer) under various values of vertical load—corresponding frequency change ratio in brackets.

	Aluminium Beam		Aluminium Beam with Polymer Layer (7 mm)	
Mass of Weight (kg)	*f*_1_ * (Hz)	*f*_2_ ** (Hz)	*f*_1_ * (Hz)	*f*_2_ ** (Hz)
1	3.1 (1.00)	37.3 (1.00)	3.0 (0.97)	32.9 (0.88)
2.5	2.1 (0.68)	36.6 (0.98)	2.0 (0.64)	31.9 (0.86)
3.5	1.8 (0.58)	36.6 (0.98)	1.7 (0.55)	31.8 (0.85)
5	1.5 (0.48)	36.6 (0.98)	1.4 (0.45)	31.8 (0.85)
6	1.4 (0.45)	36.6 (0.98)	1.3 (0.42)	31.8 (0.85)

**Table 2:** Mean values of damping ratios, corresponding to the 1st (*) and the 2nd (**) mode of free vibrations, calculated for aluminium cantilever beam (without and with polymer layer) under additional weights with different mass—corresponding damping change ratio in brackets.

	Aluminium Beam		Aluminium Beam with Polymer Layer (7 mm)	
Mass of Weight (kg)	ζ_1_ * (%)	ζ_2_ ** (%)	ζ_1_ * (%)	ζ_2_ ** (%)
1	0.27 (1.00)	0.48 (1.00)	0.92 (3.41)	1.44 (3.00)
2.5	0.33 (1.22)	0.36 (0.75)	0.69 (2.55)	1.23 (2.56)
3.5	0.30 (1.11)	0.54 (1.12)	0.77 (2.85)	1.03 (2.14)
5	0.38 (1.41)	0.45 (0.94)	1.52 (5.63)	1.60 (3.33)
6	0.38 (1.41)	0.34 (0.71)	1.47 (5.44)	1.41 (2.94)

**Table 3:** Mean values of natural frequencies corresponding to the first two modes of free vibration calculated for composite aluminium cantilever beams (with different polymer adhesive thicknesses) under additional weights of different mass.

	Aluminium Beam with Polymer Adhesive									
Mass of Weight (kg)	0.5 mm		1.2 mm		1.75 mm		3.1 mm		5 mm	
	*f*_1_ * (Hz)	*f*_2_ ** (Hz)	*f*_1_ * (Hz)	*f*_2_ ** (Hz)	*f*_1_ * (Hz)	*f*_2_ ** (Hz)	*f*_1_ * (Hz)	*f*_2_ ** (Hz)	*f*_1_ * (Hz)	*f*_2_ ** (Hz)
0	7.7	40.1	8.3	39.9	8.2	38.3	8.0	35.9	7.5	34.1
1	5.3	40.2	5.6	40.1	5.6	38.5	5.3	36.0	5.1	34.2
2.5	4.0	41.6	4.3	40.7	4.3	39.2	4.2	37.7	4.0	30.6
3.5	3.5	38.4	3.7	41.4	3.7	40.2	3.7	33.5	3.5	31.9
5	3.1	38.6	3.2	37.8	3.3	36.1	3.2	35.0	3.1	32.5
6	2.8	38.6	3.0	38.3	3.0	36.2	3.0	35.6	2.8	32.1

* in relation to the 1st mode of free vibration. ** in relation to the 2nd mode of free vibration.

**Table 4:** Mean values of damping ratios, corresponding to the first (*) and the second (**) mode of free vibrations for composite aluminium cantilever beams (with different polymer adhesive thicknesses) under additional weights with differing mass.

	Aluminium Beam with Polymer Adhesive									
Mass of Weight (kg)	0.5 mm		1.2 mm		1.75 mm		3.1 mm		5 mm	
	ζ_1_ * (%)	ζ_2_ ** (%)	ζ_1_ * (%)	ζ_2_ ** (%)	ζ_1_ * (%)	ζ_2_ ** (%)	ζ_1_ * (%)	ζ_2_ ** (%)	ζ_1_ * (%)	ζ_2_ ** (%)
0	0.66	1.37	0.72	1.29	0.98	1.30	1.31	1.11	1.01	1.01
1	0.78	1.43	0.93	1.27	1.06	1.39	1.22	1.28	1.29	1.06
2.5	0.45	2.39	0.75	1.78	0.89	1.41	1.07	2.45	1.31	2.27
3.5	0.60	1.98	0.84	2.97	0.94	2.77	1.09	2.11	1.31	1.68
5	0.50	1.52	0.80	2.80	0.88	2.65	1.23	2.03	1.65	1.48
6	0.55	1.36	0.97	2.23	0.92	1.91	1.29	1.62	1.49	1.17

**Table 5:** Values of the mass change factor α and the change in damping ratio Δ*ζ* determined for the ratio of evenly distributed masses μ_AP_/μ_A_ of the plain aluminium cantilever beam and of the aluminium cantilever beam with the polymer layer of thickness 7 mm.

	Change in Mass from Aluminium Beam to Aluminium Beam with Polymer Adhesive for Thickness Increment from 0 mm to 7 mm	
Mass of Weight (kg)	7 mm	
	α (-)	Δζ * (-)
0	1.288	0.473
1	1.119	0.326
2.5	1.063	0.244
3.5	1.048	0.214
5	1.036	0.185
6	1.030	0.171

* in relation to the 1st mode of free vibration.

**Table 6:** Values of the mass change factor α and the change in damping ratio Δ*ζ* determined for the ratio of evenly distributed masses μ_AP_/μ_A_ of the aluminium cantilever beam with a polymer layer of various thickness increments from 0.7 mm to 4.5 mm.

	Change in Mass for Aluminium Beam with Polymer Adhesive for Thickness Increment from 0.7 mm to 4.5 mm							
Mass of Weight (kg)	0.7 mm		1.25 mm		2.6 mm		4.5 mm	
	α (-)	Δζ * (-)	α (-)	Δζ * (-)	α (-)	Δζ * (-)	α (-)	Δζ * (-)
0	1.011	0.106	1.020	0.141	1.042	0.202	1.073	0.262
1	1.005	0.074	1.010	0.098	1.020	0.141	1.035	0.184
2.5	1.003	0.055	1.005	0.074	1.011	0.106	1.020	0.139
3.5	1.002	0.049	1.004	0.065	1.009	0.094	1.015	0.123
5	1.002	0.042	1.003	0.056	1.007	0.081	1.011	0.106
6	1.002	0.039	1.003	0.052	1.006	0.075	1.010	0.098

* in relation to the 1st mode of free vibration.
